# Establishment of a hTfR mAb-functionalized HPPS theranostic nanoplatform

**DOI:** 10.7150/ntno.41741

**Published:** 2020-03-26

**Authors:** Qi He, Zilong Guo, Mingpeng Fu, Hongling Tang, Huifen Zhu, Guanxin Shen, Yong He, Ping Lei

**Affiliations:** 1 Department of Immunology, School of Basic Medicine, Tongji Medical College, Huazhong University of Science and Technology, Wuhan, China.; 2 Department of Nuclear Medicine and PET Center, Zhongnan Hospital of Wuhan University, Wuhan, China.; 3 Department of Transfusion Medicine, Shandong Provincial Hospital affiliated to Shandong First Medical University, Jinan, China.

**Keywords:** transferrin receptor, antibody, nanocarrier, active targeting, delivery

## Abstract

**Rational**: Many efforts have been made to develop ligand-directed nanotheranostics in cancer management which could afford both therapeutic and diagnostic functions as well as tumor-tailored targeting. Theranostic nanoplatform targeting transferrin receptor (TfR) is an effective system for favorable delivery of diagnostic and therapeutic agents to malignancy site.

**Methods**: To enable amalgamation of therapy and diagnosis to many TfR^+^ tumor, hTfR (human TfR) monoclonal antibody (mAb)-functionalized HPPS nanoparticle (HPPS-mAb) was prepared with hTfR mAb on the shell and with fluorophore DiR-BOA in the core. The targeting specificity was investigated* in vitro* by immunostaining and *in vivo* using a double-tumor-engrafted mouse model. HPPS-mAb/siRNA effect on HepG2 cells was determined by RT-PCR and western blot.

**Results**: HPPS-mAb could specifically target cancer cells through TfR and achieve tumor accumulation at an early valuable time node, thus efficiently delivering therapeutic survivin siRNA into TfR^+^ HepG2 cells and mediating cell apoptosis. DiR-BOA can act as an imaging tool to diagnose cancer.

**Conclusions**: Our studies provide a promising TfR mAb-directed theranostic nanoplatform candidate in tumor molecular imaging and in TfR targeted tumor therapy.

## Introduction

An optimized cancer treatment strategy would deliver the right type of therapeutic drugs to the right target, to achieve localized control of the disease efficiently with minimal systemic toxicity. Emerging cancer nanotheranostics offers promising nanoplatforms with diversified capabilities for drug loading and release, for tumor targeting and imaging, and eventually for monitoring cancer therapy non-invasively and in real time. A traditional nanotheranostic agent is integrated with both diagnostic and therapeutic moieties, and is often coupled with a ligand for active targeting 1.

The widely exploited nanomaterial for cancer nanomedicine research is a liposome which is loaded by doxorubicin drugs to make the first FDA approved nano drug Doxil™/Caelyx™ 2. Other several nanomedicines, such as Abraxane™, GenexolPM™, and Onivyde™ have been approved for clinical use for cancer treatment 3. However, the development of the nanomedicines has been slow because of a great deal of limitations. For example, gold particles and quantum dots have their inherent cytotoxicities that go against with high dosage use 4. Some liposomal drugs exhibit biotoxicity or result in “complement activation-related pseudoallergy” (CARPA) via intravenous injection 5. Endogenous high density lipoprotein (HDL) as a nanocarrier has drawn attention for its complete biodegradation, non-immunogenicity and scarce reticuloendothelial system (RES) uptake 6. HDL-like peptide- phospholipid nanoparticle (HPPS) mimics the structural and functional properties of plasma- derived HDL 7, 8. Moreover, HPPS has the ability to self-assemble cholesterol, thus, it could be an efficient non-viral vector to load chol-modified nucleic acid molecules 9.

Tumor-tailored targeting approaches include active targeting. Drug-loaded nanoparticles modified with specific ligands can bind to receptors on the surface of tumor cells, leading to the accumulation of nanoparticles on the surface of certain cells 10. Transferrin receptor (TfR, CD71), a type II transmembrane glycoprotein, is involved in cell iron uptake in the course of cell growth and proliferation 11. TfR has been found highly expressed in many malignant tumors but maintains a low expression level in normal cells 12-14. Besides, TfR is an endocytic receptor which allows internalization of iron-bound transferrin (Tf). Published reports and we confirmed that antibody against TfR also induced TfR endocytosis followed by transport to lysosomal compartments 15, 16. This cellular uptake pathway and overexpression on tumor cells make TfR one of the most exploited target spots in tumor targeted therapy 17-19. Our previous studies demonstrated antibody against TfR could recognize tumor cells with high efficiency *in vitro* and ^131^I-TfR Ab displayed a feature of specific accumulation in tumor tissue *in vivo* 20, 21. These TfR Ab-modified peptides, polylysine or polyethylenimine exhibit both intrinsic cytotoxic activity and the ability to deliver a wide variety of therapeutic agents into cancer cells 22-24.

In this study, we establish a TfR mAb (monoclonal antibody) functionalized nanoparticle HPPS for TfR^+^ enhanced tumor targeting. Inside the core, a lipid-anchored near-infrared fluorophore DiR-BOA (1,1'-dioctadecyl-3,3,3',3'-tetramethylindotri-carbocyanine iodide bisoleate) serves as the model drug cargo and diagnostic moieties for optical identification. Results show that HPPS-mAb possesses the ability to reinforce targeting to tumor cells and achieves preferential recognition by TfR in a complex biological environment. Furthermore, the formed nanoparticles are capable of delivering their cancer therapeutic target siRNA payloads into TfR^+^ tumor cells to substantially mediate cell apoptosis. Therefore, the HPPS-mAb is a promising theranostic nanoplatform to enable amalgamation of therapy and diagnosis to many TfR^+^ tumors.

## Materials and Methods

### Cell culture

HepG2, U87, Hela, and MDA-MB-231 tumor cells (China Center for Type Culture Collection, Wuhan, China) were cultured in DMEM complete growth medium (Gibco, Thermo Fisher Scientific, USA) supplemented with 10% FBS (Sijiqing, Hangzhou, China) and 100 U/ml penicillin, 100 μg/ml streptomycin. CHO-hTfR (human TfR) cells which stably express hTfR-GFP and control CHOvec cells which stably express vector control 16 were cultured in RPMI 1640 medium (Gibco) supplemented with 300 μg/ml Geneticin. All cells were incubated at 37 °C in a humidified atmosphere containing 5% CO_2_.

### TfR mAb preparation

The hybridoma 7579 was intraperitoneally inoculated into Balb/c (nu/nu) nude mice (female, 6w old, Beijing Huafukang Biological Technology Co. Ltd, Beijing, China) to produce ascites for the preparation of monoclonal antibody against transferrin receptor (TfR mAb) as described before 20. The antibodies were purified from the ascites using caprylic acid and 50% ammonium sulfate precipitation. Then the precipitate was identified by SDS-PAGE (sodium dodecyl sulfate-polyacrylamide gel electrophoresis) and immunofluorescence assay. The purity was estimated based on the integrated density of protein bands on SDS-PAGE gel picture using Image J.

### Nanoparticles preparation

DiR-BOA-loaded HPPS was kindly provided by Professor Zhang Zhihong (Huazhong University of Science and Technology, Wuhan, China). HPPS was prepared firstly through a lipid emulsion film formation with DMPC (1,2-dimyristoyl-sn-glycero-3-phosphocholin), DSPE-PEG2000 (1,2-distearoyl-sn-glycero-3-phosphoethanolamine-polyethylene glycol 2000) maleimide, cholesterol oleate, and DiR-BOA. Then the lipid emulsion film was titrated with 18aa apoA-1 mimetic peptide (AP) solution to form spherical complete nanoparticle HPPS. Sulfhydryl modification of mAb and isotype (mouse polyclonal IgG, Yeasen, Shanghai, China) was achieved using thiolating reagent 2-Iminiothiolane (Traut's, Sigma, USA) to crosslink with maleimide functionalized HPPS 7, 16. The generated HPPS-mAb or HPPS-isotype was purified by fast protein liquid chromatography (FPLC) (AKTA, Amersham Biosciences, USA).

### Characterization of nanoparticles

Transmission electron microscopy (TEM) was performed to determine the size dispersion and morphology of nanoparticles. The particle hydrodynamic diameter distributions were measured by dynamic light-scattering (DLS) using Zetasizer (Nano-ZS90, Malvern Instruments, UK). The potential was measured using M3-PALS technology (Zetasizer Nano-ZS90). DiR-BOA concentration of nanoparticles was determined by a standard curve of DiR-BOA via detecting its absorption at 700 nm wavelength.

### Confocal microscopy

CHO-hTfR cells were cocultured with nanoparticles (DiR-BOA 1 μM) for 30 min at 37 °C. The imaging was implemented through a Zeiss LSM710 confocal laser scanning microscopy (Zeiss, Oberkochen, Germany) with excitation at 488 nm for EGFP (Enhanced Green Fluorescent Protein) and 633 nm for DiR-BOA. Images were acquired and analyzed using Zen-2009 software.

### Flow cytometry

The incubation concentration consistency of HPPS and HPPS-mAb was referred to DiR-BOA determined by a standard curve. 3×10^5^ cells (including HepG2, U87, Hela, MDA-MB-231, and CHOvec, CHO-hTfR) were incubated with different DiR-BOA concentrations of nanoparticles (10 μM~18 nM) for 30 min at 37 °C. After 3 times rinse, DiR-BOA^+^ cells were measured by flow cytometry (LSRII, BD Biosciences, USA) using APC-Cy7 color substitution excited at 633 nm. For HDL blockade assay, HepG2 cells were treated with 50 molar excess human HDL (Yeasen, Shanghai, China) for 15 min, then were incubated with HPPS-mAb or HPPS-isotype (DiR- BOA 10 μM). For apoptosis assay, HepG2 cells were stained with Annexin V FITC and PI (BD Bioscience, USA) according to the manufacturer's specifications. Apoptotic cells were analyzed by flow cytometry.

### Whole-body fluorescence imaging

All animal experiments were approved by the Ethics Committee of Tongji Medical College of Huazhong University of Science and Technology in compliance with guidelines approved by Animal Care Committee. Balb/c nude mice (female, 4-5 w old, Beijing Huafukang Biological Technology Co. Ltd, Beijing, China) were subcutaneously inoculated with 1×10^7^/ml cells (in 50 μl) mixed with matrigel (BD Biosciences, CA, USA). CHOvec cells were inoculated in the left hind flanks and CHO-hTfR cells in the right hind flanks. 7-14 days later, when tumor size (the largest diameter) of any side reached 10mm, tumor-bearing mice (four in each group randomized) were intravenously injected with nanoparticles at a DiR-BOA dose of 10 nmol via the tail vein. Whole-body fluorescence images were taken with a CRI Maestro *in vivo* imaging system at 12 h, 24 h post injection. The excitation wavelength of HPPS contained DiR-BOA was 700 nm and the emission filter was 750 nm long pass.

### Preparation of HPPS-mAb/siRNA

Cholesterol modified survivin-siRNA (chol-si-survivin, Genepharma, Shanghai, China) consisted of the sense strand 5'-chol-AGCAUUCGUCCGGUUGCGCsTsT-3' and antisense strand 5'-GCGCAACCGGACGAAUGCUTsT-3'. Cholesterol-conjugated siRNA bearing a scrambled sequence (chol-si-NC) consisted of the sense strand 5'-chol-UUCUCCGAACGUGUCACGUsTsT-3' and the antisense strand 5'-ACGUGACACGUUCGGAGAATsT-3'. Chol-siRNA and HPPS-mAb were mixed at 1:1, 1:5, 1:10, 1:20, 1:30 molar ratio (siRNA: DiR-BOA) at RT for 30 min to obtain HPPS-mAb/siRNA. Then the HPPS-mAb/siRNA was loaded into 2% agrose gel containing nucleic acid dye. Naked siRNA and HPPS-mAb were mixed and run at molar ratio 1:5 using the same experimental condition.

### RT-PCR

HepG2 cells were treated with HPPS-isotype/ siRNA and HPPS-mAb/siRNA for 48 h. Then cells were lyzed using Trizol (Thermo Fisher Scientific, USA) according to manufacturer's recommendation. The achieved mRNA was reverse transcribed to cDNA and amplified as previously reported 25. Agarose gel electrophoresis was used to detect the PCR reaction product and results were quantified by Image J software.

### Western blotting

HepG2 cell lysates were separated by 12% SDS- PAGE and transferred to polyvinylidene difluoride (PVDF) membrane. After blockade, membrane was probed with rabbit anti-survivin polyclonal antibody (Proteintech Group, Wuhan, China) followed by HRP-labeled goat anti rabbit antibody (Santa Cruz, CA, USA). Bands were detected using ECL kit (Pierce, Rockford, USA). β-actin was set as loading control. Blank control referred to PBS.

### Statistical analysis

The uptake of nanoparticles was analyzed by two-way ANOVA followed by LSD post hoc test. Survivin mRNA or protein expression was analyzed by one-way ANOVA followed by Bonferroni post hoc test. ζ-potential was analyzed by student's two-sided t-test using SPSS 17.0 statistical software (IBM, USA). All values in the study were expressed as means ± SD. Differences were considered significant statistically as *P* < 0.05.

## Results

### Characteristics of HPPS-mAb nanoparticles

**Figure 1:**
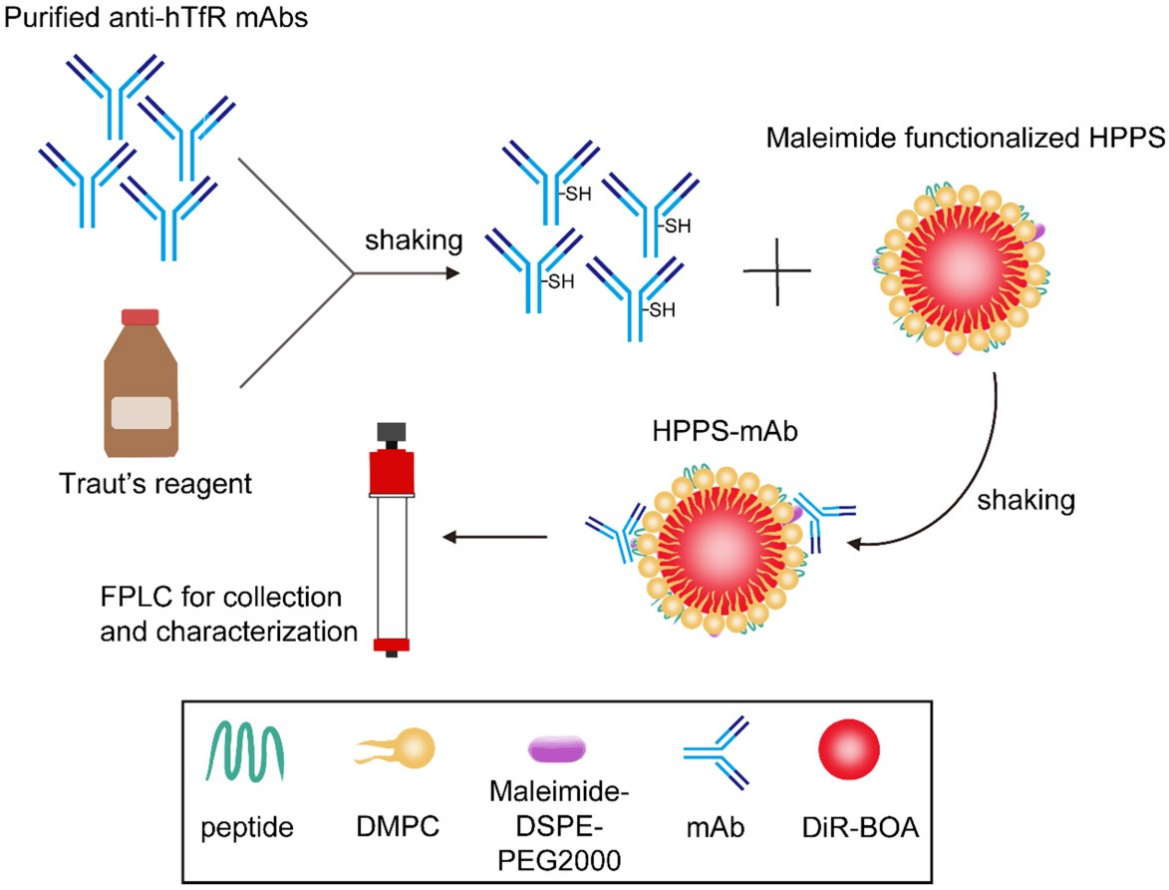
A schematic diagram of TfR mAb coupling process with HPPS nanoparticle (The maleimide functionalized HPPS was kindly provided by our cooperative laboratory). HPPS: HDL-like peptide-phospholipid nanoparticles.

**Figure 2:**
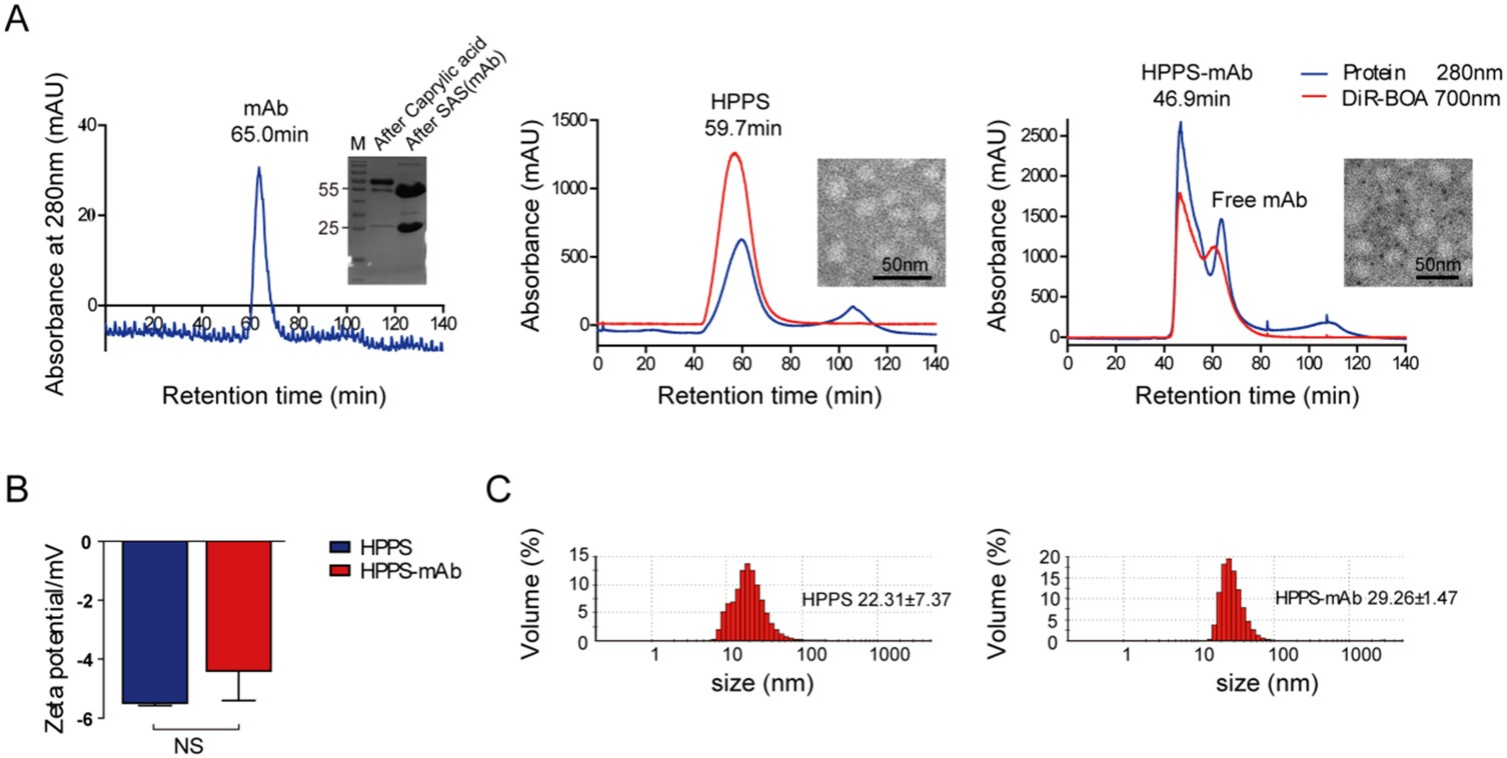
The purification and characterization of mAb functionalized HPPS. **(A)** Purification profiles of mAb (left), HPPS (middle) and HPPS-mAb (right) by FPLC. Inset: SDS-PAGE of purified mAb (left) and TEM image of HPPS (middle) and HPPS-mAb (right) with approximate size. **(B)** ζ-potential change and size shift **(C)** of the HPPS particles after loading with TfR mAb. Student's t-test was used for statistical comparisons, *P* < 0.05. FPLC: fast protein liquid chromatography; HPPS: HDL-like peptide-phospholipid nanoparticles; mAb: monoclonal antibody; SDS-PAGE: sodium dodecyl sulfate, polyacrylamide gel electrophoresis; TEM: transmission electron microscopy; TfR: transferrin receptor.

HPPS mainly contained lipids including DMPC, DSPE-PEG2000, CO (cholesterol oleate) and 18aa ApoA-1 mimetic peptide (AP). TfR mAb was isolated from ascites with purity of about 98%. The mAb was thiol-functionalized and conjugated to the surface of HPPS to obtain HPPS-mAb. DiR-BOA, a near-infrared fluorescent dye, which is a small hydrophobic molecule fluorophore amenable to bisoleoyl-based nanoparticle incorporation, has been loaded into the core of HPPS as a model functional cargo. A concise synthetic process was shown in Figure 1. The nanoparticles used in coupling were always from one and the same batch with the original HPPS. The fast protein liquid chromatography (FPLC) profiles showed mAb, HPPS and HPPS-mAb with different absorption peak time. Nanoparticles with larger size have an earlier peak time. For instance, the peak time of free mAb was around 65 min and of HPPS crosslinked mAb was around 46.9 min. The retention time of HPPS-mAb shifted earlier than that of TfR mAb. And the peak time of HPPS-mAb was also distinctly different from the one of HPPS, which indicated that at least one mAb had been coupled with HPPS to give a larger particle formation of HPPS-mAb. The protein curve of HPPS-mAb described a similar trend of fluctuations with DiR-BOA payload between 45-55 min which was identified as successful coupling HPPS-mAb. As for the absorbance peaks at 280 nm in analysis using non-modified HPPS nanoparticle, the one appearing at 59.7 min was HPPS-incorporated AP and the other at 100 min was free AP due to excess AP added during HPPS synthesis. The absorbance peak (at 700 nm) at fraction between 60-70 min in HPPS-mAb was unconjugated HPPS due to excess HPPS added during synthesis. Hence, the utmostly pure HPPS-mAb was obtained by collecting fractions between 45-55 min (Figure 2A).

ζ-potential measurements showed that loading mAb on HPPS did not significantly change surface charge of the nanoparticles (Figure 2B). Dynamic light scattering (DLS) and transmission electron microscopy (TEM) revealed the hydrodynamic diameter of this spherical, relatively homogeneous HPPS-mAb to be 29.26 ± 1.47 nm and HPPS to be 22.31 ± 7.37 nm (Figure 2C). These 30 nm nanoparticles were reported to be able to diffuse into the central areas of tumors, leading to the best treatment outcome 26. All these data suggested that mAb conjugation did not significantly disrupt the integrity of the HPPS nanoparticles.

### TfR targeting of HPPS-mAb *in vitro*

**Figure 3:**
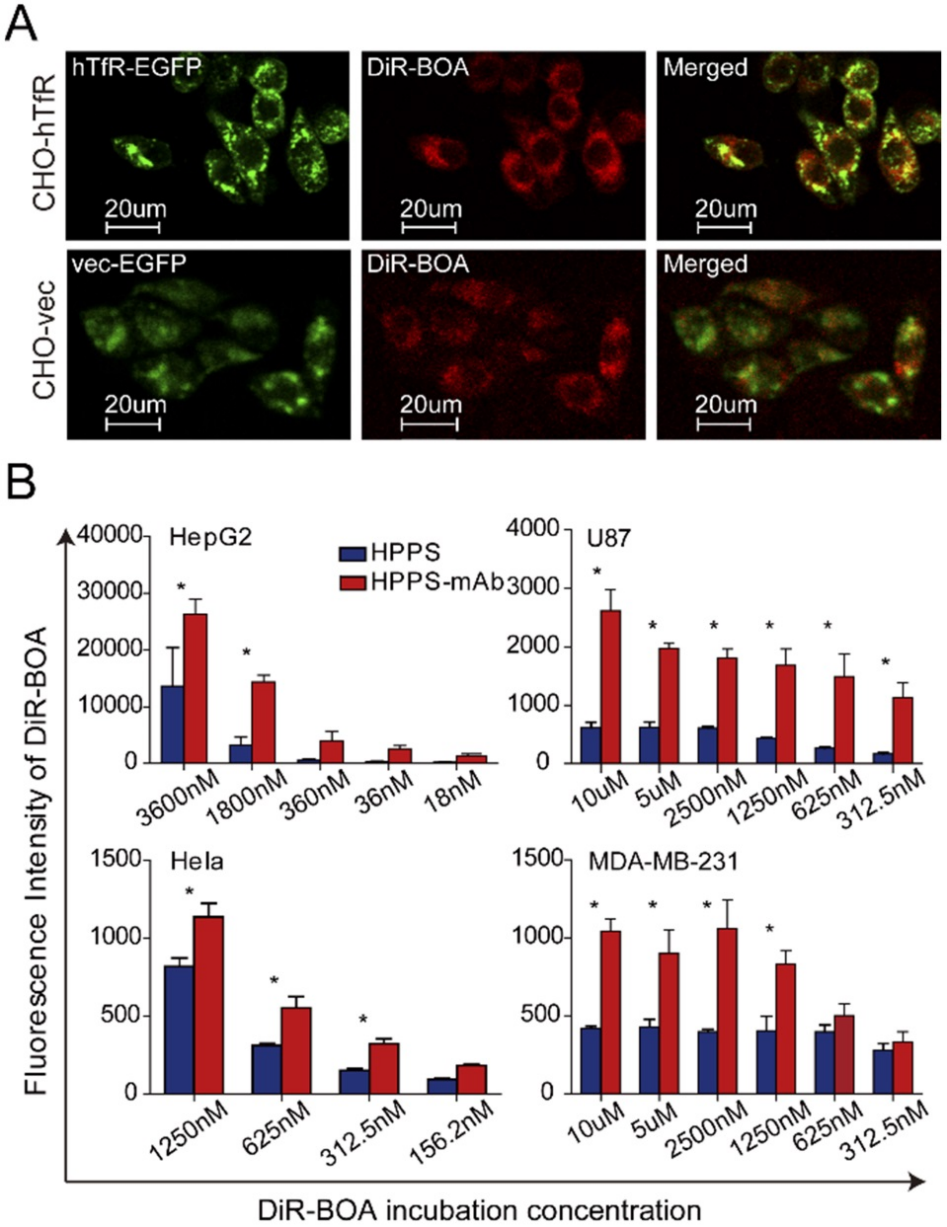
Targeting of DiR-BOA bearing HPPS-mAb. **(A)** Confocal imaging of red DiR-BOA fluorescence (cargos) in CHO-hTfR and CHOvec cells, respectively. **(B)** Uptake of nanoparticles by TfR positive HepG2, Hela, U87, and MDA-MB-231 cells. The cellular uptake of nanoparticles was positively correlated with the fluorescence intensity of DiR-BOA. Mean values ± standard deviation, n = 3. Two-way ANOVA with LSD post hoc analysis was used for statistical comparisons, *P < 0.05. CHO-hTfR: CHO cells stably expressing human TfR; CHOvec: CHO cells stably expressing vector control; DiR-BOA, 1,1'-dioctadecyl-3,3,3',3'- tetramethylindotri-carbocyanine iodide bisoleate; HPPS: HDL-like peptide-phospholipid nanoparticles; TfR mAb: transferrin receptor monoclonal antibody.

The molar concentration of DiR-BOA was employed to normalize the nanoparticle quantification. DiR-BOA dose referred to the concentration within the nanoparticles. To confirm the targeting specificity of HPPS-mAb, a plasmid encoding a hTfR-EGFP fusion protein was stably transfected into hTfR-negative CHO cells to establish CHO-hTfR cells, and the empty plasmid was treated the same to establish CHOvec cells (establishment of CHO-hTfR and CHOvec cell strains were described in 16). The CHO-hTfR cells had similar hTfR expression and functionality with HepG2 cells which naturally express hTfR. We previously reported that hTfR mAb-conjugated HPPS nanoparticles could be preferentially taken up by CHO-hTfR cells and this uptake was hTfR targeting 16. In this article, based on the DiR-BOA fluorescence signal, the confocal microscopy confirmed that EGFP expressing cells had significant HPPS-mAb uptake. Orange to yellow overlap fluorescence suggested the colocalization of hTfR-EGFP and DiR-BOA on the periphery and in the perinuclear regions of the cell. This demonstrated that HPPS-mAb could specifically deliver its cargo (DiR-BOA) into target cells via TfR-mediated endocytosis (Figure 3A). Vector control CHOvec cells were found also to take up the DiR-BOA fluorescence which was evenly diffused in cells. Given that class B scavenger receptor SR-BI is an HDL receptor 27, HPPS moiety could contact with widely expressing SR-BI and release DiR-BOA to the cytoplasm in a SR-BI dependent manner 28. Hence this indicated that SR-BI took responsibility for natural HPPS-mAb uptake in CHOvec and for partial non-colocalized red fluorescence in CHO-hTfR cells.

After confirming the specific uptake of HPPS-mAb by TfR-mediated endocytosis, we evaluated the performance of HPPS-mAb in various TfR-expressing tumor cell lines, including HepG2, Hela, U87, and MDA-MB-231 cells. Figure 3B showed that DiR-BOA was accumulated in both HPPS and HPPS-mAb treated cancer cells. And as the concentration of nanoparticles increased, the amount of DiR-BOA uptake also increased. However, these cells showed a higher uptake of HPPS-mAb than of HPPS. As for Hela, HPPS-mAb showed at least 1.4 folds fluorescence increase. In U87, HPPS-mAb uptake was 4-5 times more than HPPS. It suggested that mAb conjugation provided HPPS-mAb dominance of nanoparticle intake in these tumor cell lines despite varying expression levels of TfR. These data demonstrated that TfR mAb conjugation had the ability to enhance the uptake of HPPS-mAb.

**Figure 4:**
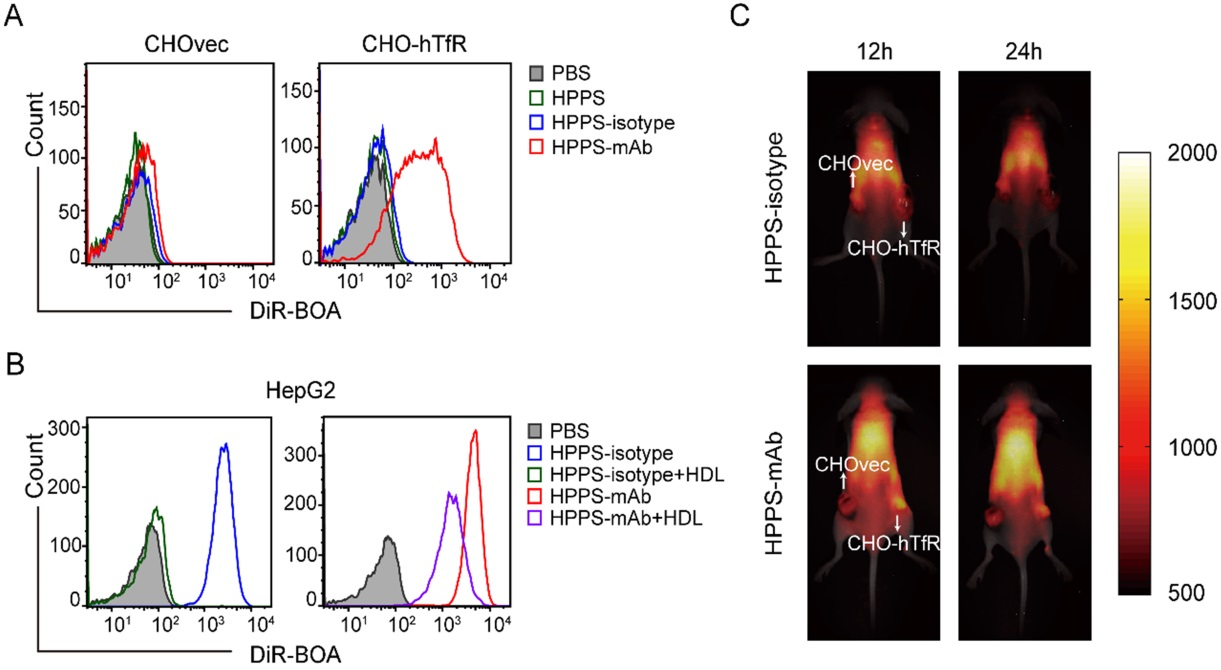
Coordinated targeting of HPPS-mAb *in vitro* and *in vivo*. **(A)** Representative FCM histograms showed the cellular uptake of three nanoparticles at DiR-BOA concentration 312.5 nm. **(B)** HepG2 cells were incubated with excess HDL for 15 min. Then cells were treated with HPPS-isotype and HPPS-mAb (10 μM DiR-BOA concentration). Representative FCM histograms showed the uptake of HPPS-mAb. **(C)** A double-engrafted tumor mouse model was established with CHOvec (TfR^-^) on the left flank and CHO-hTfR (TfR^+^) on the right flank. *In vivo* whole body imaging at two time points was shown. Top row: injected with HPPS-isotype; bottom row: injected with HPPS-mAb. The white arrows refer to the location of xenografts. n = 4. CHO-hTfR: CHO cells stably expressing human TfR; CHOvec: CHO cells stably expressing vector control; DiR-BOA, 1,1'-dioctadecyl-3,3,3',3'- tetramethylindotri-carbocyanine iodide bisoleate; FCM: flow cytometry; HPPS-mAb: HDL-like peptide-phospholipid nanoparticles conjugated with TfR mAb.

To further understand the TfR specificity of HPPS-mAb delivery, HPPS-isotype nanoparticles were constructed using mouse polyclonal IgG under the same synthesis procedure. CHO-hTfR and CHOvec cells were used as hTfR^+^ and hTfR^-^ contrast. As expected, both CHOvec and CHO-hTfR exhibited low uptake of HPPS and HPPS-isotype. For HPPS-mAb, CHO-hTfR showed higher DiR-BOA fluorescence intensity than TfR^-^ CHOvec cells (25 folds) which indicated TfR targeting specificity of HPPS-mAb (Figure 4A). To further corroborate TfR targeting of HPPS-mAb, excess human HDL was used to block SR-BI mediated nanoparticle endocytosis in SR-BI^+^ HepG2 cells. In Figure 4B, HDL blockade could inhibit the uptake of HPPS-isotype completely but partially of HPPS-mAb. Accordingly, the outermost mAb on HPPS-mAb must be the first contact with cell membrane and mAb-TfR interaction could give full play to active targeting of HPPS-mAb.

### TfR targeting of HPPS-mAb *in vivo*

In order to investigate the *in vivo* functional performance of HPPS-mAb, a double-tumor-engrafted mouse model was introduced with CHOvec (TfR^-^) on the left flank and CHO-hTfR (TfR^+^) on the right flank. HPPS-mAb and HPPS-isotype were injected via tail vein to compare the particles accumulation. Benefiting from its excellent penetrability, DiR-BOA was allowed for detection of nanoparticle internalization and tumor visualization. In Figure 4C, *in vivo* whole body images showed that loading with DiR-BOA in the core of nanoparticle, HPPS-mAb nanoparticles were observed to accumulate and DiR-BOA fluorescence signals were found to peak in CHO-hTfR tumor tissues as early as 12 h after systemic administration. The HPPS-mAb showed different distribution pattern between TfR^+^ and TfR^-^ tissues. A direct superior accumulation of DiR-BOA meant that it was distributed to its target tumor tissues more quickly than to non-target tumor tissues. By contrast, HPPS-isotype rarely accumulated in bilateral xenografts at both time points. Taken together, these data demonstrated that HPPS-mAb can be targeted specifically to cancer cells through TfR at an early valuable node, and DiR-BOA can act as an imaging tool to diagnose cancer and to monitor therapy non-invasively and in real time.

### HPPS-mAb mediated RNA interference

**Figure 5:**
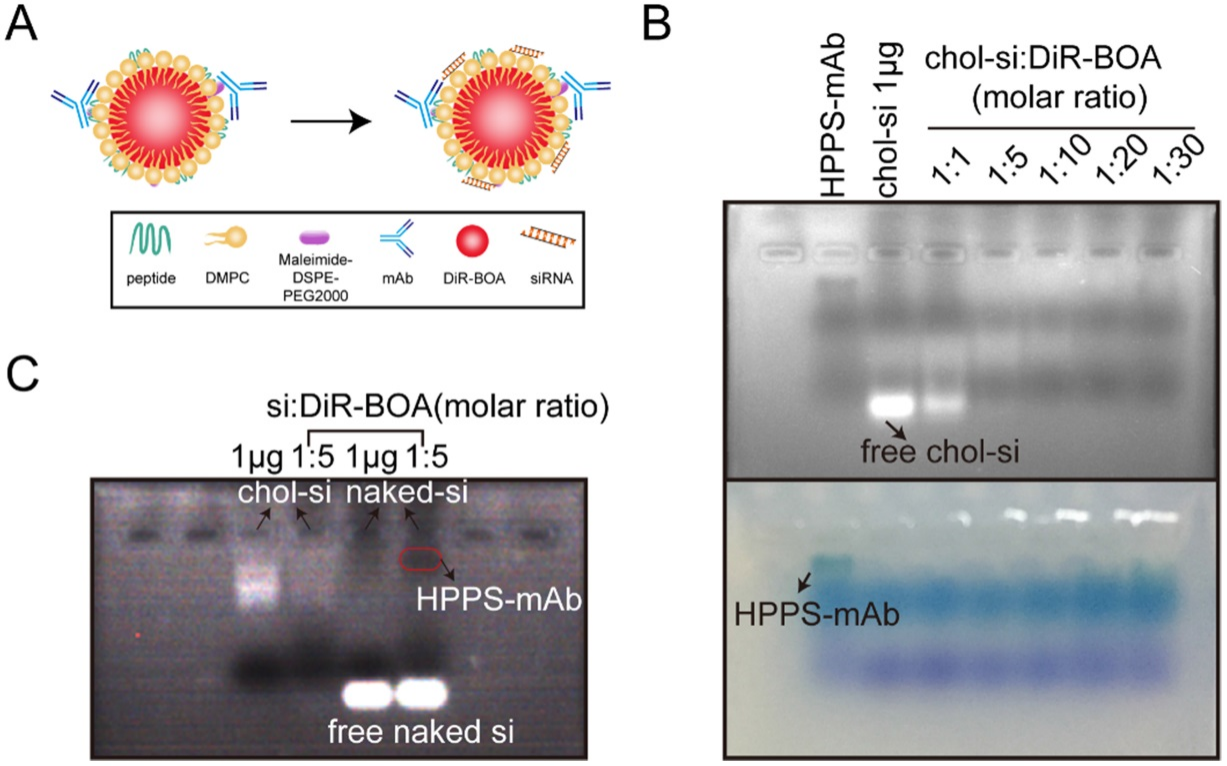
Validation of HPPS-mAb/siRNA. **(A)** Design of HPPS-mAb/siRNA with fluorophores in the core. **(B)** UV illumination and bright image showed the best conjugation ratio of chol-siRNA with HPPS-mAb (DiR-BOA) through gel shift assay. **(C)** Naked siRNA showed no conjugation with HPPS-mAb. Chol-si = 1 µg. Chol: cholesterol-modified; DiR-BOA, 1,1'-dioctadecyl-3,3,3',3'- tetramethylindotri-carbocyanine iodide bisoleate; HPPS-mAb/siRNA: HDL-like peptide-phospholipid nanoparticles conjugated with TfR mAb and loaded with siRNA; si: siRNA.

After verifying that HPPS-mAb nanoparticles could deliver DiR-BOA cargo specifically into TfR^+^ tumor *in vivo*, HPPS-mAb was incorporated by other hydrophobic therapeutic payloads to test its potential suitability for the targeted delivery of clinical relevant cancer therapeutics. Since the naked siRNA will be eliminated rapidly post intravenously injection as a result of kidney filtration and/or serum degradation, HPPS-mAb was loaded with cholesterol-modified survivin siRNA (as shown in the schematic plot in Figure 5A) to assess the targeted siRNA delivery. Different molar ratios of siRNA:DiR-BOA were prepared to obtain the best mixture ratio avoiding free siRNA wandering. Figure 5B showed that HPPS-mAb was successfully loaded with chol-siRNA. The optimizing molar ratio of choi-si to HPPS-mAb (DiR-BOA) was set at 1:5 (Figure 5C).

**Figure 6:**
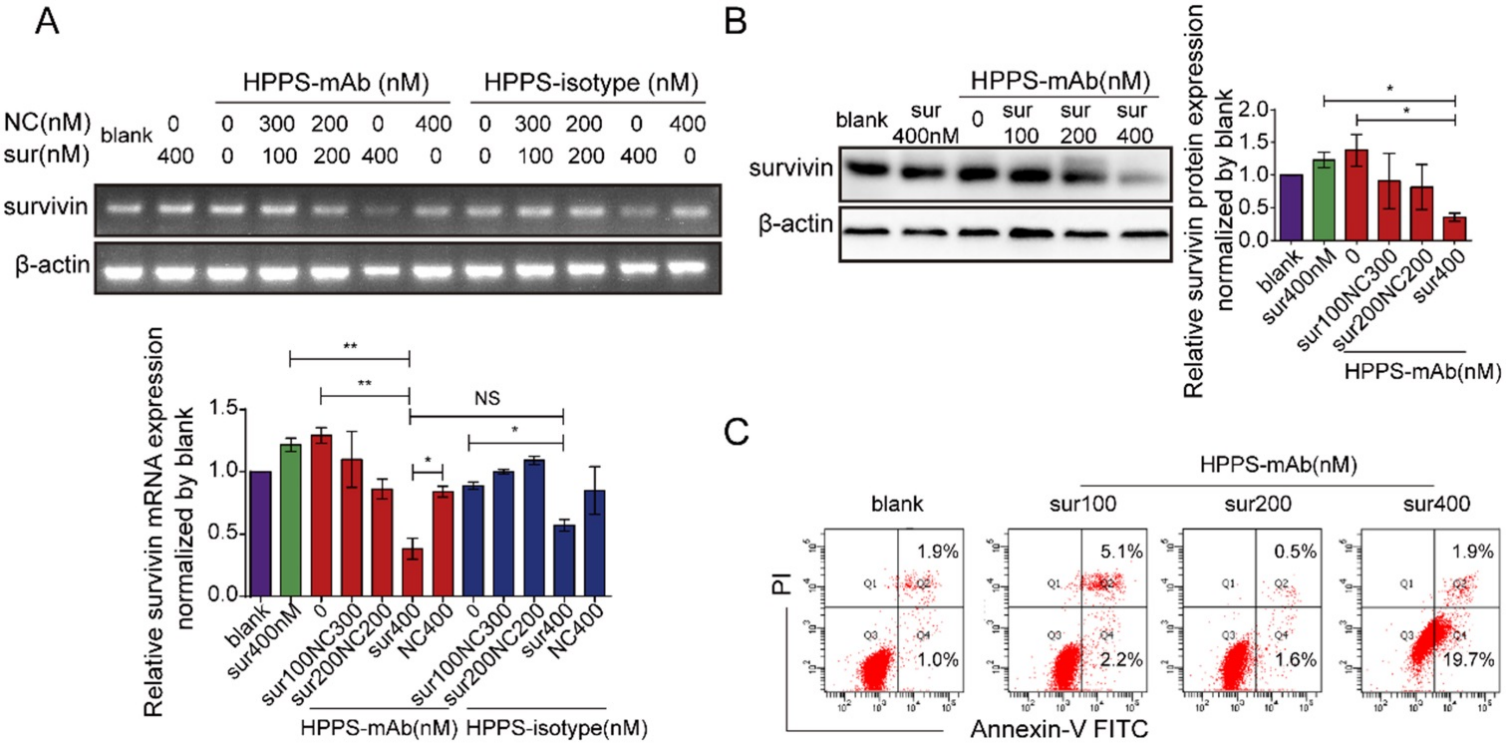
*In vitro* knock-down efficacy of HPPS-mAb/siRNA in HepG2 cells. Survivin expression in RNA level **(A)** and in protein level **(B)** in HepG2 cells treated with HPPS-mAb/siRNA for 48h. Intensity analysis was represented as mean ± SD. n = 3, one-way ANOVA with Bonferroni post hoc analysis was used for statistical comparisons, *P < 0.05, **P < 0.01. **(C)** Apoptosis assay for HPPS-mAb/siRNA treated cells by FCM. Representative FCM histograms were showed. Apoptosis rates were plotted. HPPS-mAb/siRNA: HDL-like peptide-phospholipid nanoparticles conjugated with TfR mAb and loaded with siRNA; NC: chol-si-NC; Sur: chol-si-survivin.

HPPS-mAb/siRNA treatment decreased the survivin expression at RNA level (Figure 6A) and at protein level (Figure 6B) in a dose-dependent manner in HepG2 cells. 400 nM chol-si equivalent HPPS- mAb/siRNA treatment could almost completely abrogate the survivin expression, but 400 nM chol-si-survivin alone could not. Although the survivin level got a certain degree of reduction induced by HPPS-isotype/siRNA, the reduction degree was still less than HPPS-mAb mediated RNA silencing.

To investigate the effect of siRNA accumulation in tumor cells, apoptosis of HepG2 cells were assessed 48 h after HPPS-mAb/siRNA treatment. Data showed that the HPPS-mAb/siRNA caused an induction of apoptosis in a dose dependent way. The 400 nM HPPS-mAb/siRNA could significantly induce more cells underwent apoptosis than blank control (PBS treatment) (Figure 6C).

By above, HPPS-mAb could be a favorable nanoplatform to deliver therapeutic agents into tumor cells to achieve targeted tumor chemotherapy.

## Discussion

Advancements in the development of nanotheranostics have made it possible for more accurate tumor detection, cancer staging, and real-time monitoring of cancer progression and therapeutic outcomes 1, 29. To design an active targeting theranostic nanoplatform that could be generally used to a variety of tumor tissues, the membrane TfR is exploited for the site-specific delivery of diagnostic and therapeutic agents into proliferating malignant cells that overexpress TfR. Several TfR-specific targeting ligands, such as antibodies, recombinant Tf, have been developed and used to target nanoparticle carriers to TfR 30. However, since TfR is almost saturated under physiologic conditions due to high endogenous plasma concentrations of Tf 31, Tf-targeted nanoparticle types fail to target the relevant pathway. To achieve recognition by TfR in biological milieu, in the present investigation, TfR mAb functionalized nanoparticle was developed and tested its targeting to cancer cells *in vitro* and *in vivo*. Results manifested that in serum-rich conditions, TfR mAb grafted nanoparticles showed interaction with and internalization via TfR, and reduction of uptake in human TfR-negative CHO cells. The *in vivo* imaging showed the quick distribution of HPPS-mAb from blood to TfR^+^ tumor tissues, confirming targeted delivery of TfR mAb grafted nanoparticles in a complex biological environment. This enlightened us that this distribution mechanism could enable HPPS-mAb serve as potential diagnostic agent.

Except for being functionalized by targeting moieties to avoid normal tissue side effect, ideal nanocarriers should meet the following several points theoretically 32: 1) biocompatibility and biodegradable material 33, 2) effective drug loading, 3) controllable drug release 34, 4) immunological response cover. Nanocarrier HPPS is a biomimetic lipid-protein vector with non-immunogenicity, biocompatible components, peptide-mediated structural, and functional control. HPPS particles could form sub-30-nm, monodisperse, and spherical nanostructures. These nanoparticles showed a high degree of tunability within the 10-30 nm size range by varying the amount of cargo and displayed an exceptional cargo shielding properties. Payload- bearing HPPS particles could achieve excellent stability and integrity in a body fluid environment, which benefits their applications in the complicated *in vivo* environment 9, 35. To take advantage of this established nanoparticle model, HPPS was functionalized with TfR mAb to systematically investigate its TfR specific uptake.

Data showed that the HPPS-mAb nanoparticles gained favorable tumor accumulation. However, when HPPS and HPPS-mAb were incubated with multiple tumor cells respectively, a gradient accelerating phenomenon leading to enhanced nanoparticle uptake was demonstrated, questioning whether a dual targeting cooperative mechanism existed. Given that HPPS mimics the functions of plasma-derived HDL not only in its pharmacokinetics but also in its targeting specificity against SR-BI which prominently expresses in the liver and steroidogenic cells of the adrenal and gonads especially in variety of tumors 27, we acknowledge the fact that HPPS-mAb nanoparticle deposition in tumors is not governed by only TfR active targeting, but also by SR-BI, and enhanced permeability and retention effect.

The isotype construction of HPPS-mAb benefited the HPPS-mAb *in vivo* targeting imaging research. Whole body imaging showed that within 24 h, HPPS-mAb had a preferred accumulation in CHO-hTfR bearing xenograft while the HPPS-isotype owing the same nano-dimension performance showed no preferred orientation. We previously reported that radionuclide-labeled TfR-mAb accumulated and peaked in tumor tissue 24 h after systemic administration 21. In this research, accumulation of HPPS-mAb peaked as early as 12 h which could attribute to HPPS moiety acceleration by means of either SR-BI targeted uptake or EPR effect. Thus, this superior targeting accumulation gave us inspiration prompting for the potential application of HPPS-mAb as theranostic nanoplatform 1.

Many nanoparticle-based delivery strategies have been developed for improving siRNA delivery *in vivo*. Researchers have focused their attention on siRNA biological property optimization and development of safe and effective transportation systems. Studies have been reported that the cholesterol modification of siRNA, 2'-fluoro substituted nucleic acids and phosphorothioate (PS) linkages usage would enhance potency of siRNA to resist nucleases 36. By means of active targeting moiety, nanoparticle could carry siRNA entering cytoplasm mainly via endosomal participating way. The final outcome is in lysosomes and siRNA fails to come out. To overcome this, many attempts has been made 37-39. Because of its superior siRNA loading and its stability and integrity *in vitro* and *in vivo*, HPPS becomes a safe, efficient siRNA carrier using the property of transporting intracellular active cancer agents to the cytosol without the involvement of endolysosomal trafficking 9, 40, 41. In present study, survivin-siRNA was cholesterol-modified and then embedded into HPPS-mAb. This HPPS-mAb/siRNA did serve as a TfR oriented nanocarrier to substantially silence survivin expression in tumor cells and induce cell apoptosis in biological milieu.

The variations among individuals make a single nano-formulation not applicable for all kinds of tumors. The high and broad expression of TfR in various malignant tumors makes TfR mAb- functionalized nanoparticles applicable for as many tumor types as possible. And the carrier HPPS holds great relevance as they may represent biocompatible systems with relatively simple fabrication and modification. In our lab, this HPPS-mAb nanoplatform is developed to be incorporated with more other therapeutic payloads for tailored therapy for more cancer types.

## Conclusions

This study confirmed the TfR targeting, DiR-BOA imaging and effective drug release of HPPS- mAb nanocarriers in TfR^+^ cancer cells. HPPS-mAb is a suitable theranostic nanoplatform for drug-release monitoring, imaging-guided focal therapy and post-treatment response monitoring.

## Abbreviations

Chol-siRNA: cholesterol modified siRNA
CO: cholesterol oleate
DiR-BOA: 1,1'-dioctadecyl-3,3,3',3'- tetramethylindotricarbocyanine iodide bisoleate
DLS: dynamic light-scattering
DMPC: 1,2-dimyristoyl-sn-glycero-3-phosphocholin
DSPE: 1,2-distearoyl-sn-glycero-3-phosphoethanolamine
EGFP: enhanced green fluorescent protein
FCM: flow cytometry
HDL: high density lipoprotein
HPPS: HDL-like peptide-phospholipid nanoparticles
mAb: monoclonal antibody
PEG: polyethylene glycol
SDS-PAGE: sodium dodecyl sulfate-polyacrylamide gel electrophoresis
siRNA: small interfering RNA
SR-BI: scavenger receptor class B type I
TEM: transmission electron microscopy
Tf: transferrin
TfR: transferrin receptor
